# Surgical Management of a Separated Instrument and Radicular Cyst: A Nine-Month Cone Beam Computed Tomography (CBCT) Follow-up

**DOI:** 10.7759/cureus.96940

**Published:** 2025-11-16

**Authors:** Dipti Chauhan, Hemant Yadav, Anshu Minocha, Vishal Sharma

**Affiliations:** 1 Conservative Dentistry and Endodontics, Himachal Pradesh Government Dental College & Hospital, Shimla, IND; 2 General Medicine/Surgery, Maulana Azad Medical College, New Delhi, IND

**Keywords:** biodentine, cbct, fractured instrument, guided bone regeneration, luebke–oschenbein flap, platelet-rich fibrin, radicular cyst

## Abstract

Large periapical lesions complicated by fractured instruments present a significant endodontic challenge. When such teeth serve as abutments in a fixed partial denture (FPD), orthograde retreatment may be impractical. Surgical endodontics, aided by bioactive and regenerative materials, can offer a predictable, conservative alternative.

This case report highlights a middle-aged patient who presented with dull pain and intermittent pus discharge in relation to tooth #21, previously root canal treated and restored under an 8-unit FPD (teeth #15-23). Cone-beam computed tomography (CBCT) revealed a periapical lesion (measuring 13.7 × 12.8 × 14.8 mm) with labial cortical discontinuity and a 4.7-mm separated apical fragment. Because post removal and prosthesis dismantling were neither feasible nor cost-effective, a surgical approach using a Luebke-Oschenbein limited-thickness mucoperiosteal flap was adopted. The procedure included cyst enucleation, instrument retrieval, apicoectomy with ultrasonic retropreparation, retro-sealing with Biodentine, and guided bone regeneration with advanced platelet-rich fibrin (APRF) and allograft. CBCT follow-up at six months and nine months showed progressive bone fill and graft consolidation. In conclusion, in prosthetically constrained cases, microsurgical endodontic management using bioactive and regenerative materials can predictably resolve pathology while preserving existing restorations.

## Introduction

Fractured endodontic instruments occur in approximately 0.7%-6% of cases, with incidence varying based on canal curvature and operator experience [1]. Such occurrences may compromise canal debridement and sealing, predisposing the tooth to persistent periapical inflammation [2]. When the affected tooth serves as an abutment in a fixed prosthesis, conventional orthograde retreatment can endanger adjacent abutments and lead to financial or structural complications. In such situations, microsurgical endodontic management provides a direct and biologically conservative alternative [2]. Although treatment options such as prosthesis dismantling or intentional replantation exist, they were deemed impractical in this case due to financial constraints and the risk of damaging the existing fixed partial denture (FPD).

Radicular cysts have been reported to comprise approximately 15%-20% of all endodontic periapical lesions [3]. Advances in cone-beam computed tomography (CBCT) now allow precise three-dimensional assessment of lesion extent, cortical perforation, and foreign body location [4]. Simultaneously, bioactive materials such as Biodentine and regenerative adjuncts such as autologous platelet-rich fibrin (APRF) have demonstrated improved healing outcomes. This case report documents the successful surgical management of a radicular cyst associated with a separated apical instrument, with radiographic healing evaluated using CBCT over a nine-month follow-up period [5].

## Case presentation

A 37-year-old female presented with dull pain and intermittent pus discharge in the upper anterior region for three to four months. The patient had undergone root canal treatment with post-core restorations in teeth #21 and #23 two years earlier, followed by placement of an 8-unit FPD from tooth #15 to tooth #23. Examination revealed mild tenderness on palpation and intermittent sinus discharge near tooth #21. The FPD was stable, with no mobility or periodontal pockets.

**Figure 1:**
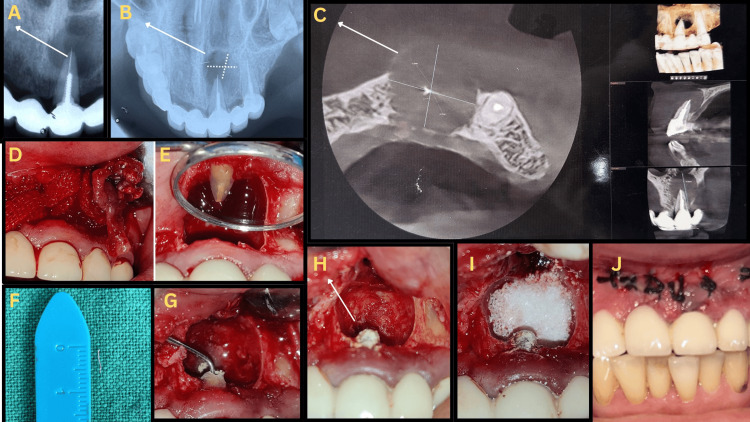
Treatment (A) Pre-operative intraoral periapical radiograph (IOPA) of tooth #21 showing a large periapical radiolucency with a separated instrument in the apical third; (B) occlusal radiograph (R/G) showing the extent of the lesion; (C) pre-operative cone-beam computed tomography (CBCT) (axial, coronal, and sagittal views) showing a 13.7 × 12.8 × 14.8 mm lesion with labial cortical discontinuity; (D) intra-operative view showing cyst enucleation; (E) visualized separated fragment; (F) retrieval of the fractured fragment; (G) apicoectomy and ultrasonic (US) retropreparation; (H) placement of Biodentine as the retrograde filling material; (I) placement of autologous platelet-rich fibrin (APRF) and allograft; and (J) suturing completed.

Pre-operative intraoral periapical radiograph (IOPA; Figure 1, Panel A) and occlusal views (Figure 1, Panel B) revealed a large periapical radiolucency. CBCT (Figure 1, Panel ​​​​​​​C) showed a well-defined lesion (13.7 × 12.8 × 14.8 mm) with loss of labial cortical plate continuity and a 4.7-mm separated fragment in the apical third of tooth #21. Orthograde retreatment was ruled out because removing the post would require dismantling the long-span FPD, risking abutment damage and significant expense. Based on lesion size, prosthetic constraints, and apical fragment position, a surgical retrograde approach was selected - consistent with current recommendations favoring microsurgical management when orthograde retrieval is impractical [6,7].

After administration of local anesthesia, a Luebke-Oschenbein limited-thickness mucoperiosteal flap was designed to preserve the marginal gingiva and avoid trauma to the prosthesis [8]. Following flap reflection, complete enucleation of the cystic lining was performed (Figure 1, Panel A), and the specimen was measured and sent for histopathology. The separated instrument was visualized and retrieved (Figure 1, Panel ​​​​​​​D). Apicoectomy of tooth #21 was carried out, and ultrasonic retropreparation was done to a depth of 3 mm (Figure 1, Panel ​​​​​​​F). The root end was sealed with Biodentine (Septodont, France), as shown in Figure 1 (Panel ​​​​​​​G). The periapical defect was packed with APRF and demineralized bone allograft (Figure 1, Panel ​​​​​​​H). Primary closure was achieved using 4-0 sutures. An immediate postoperative radiograph confirmed complete retrieval and retrofill adaptation. Histopathologic examination revealed a cystic lumen lined by non-keratinized stratified squamous epithelium with dense chronic inflammatory infiltrate, confirming a radicular cyst.

**Figure 2:**
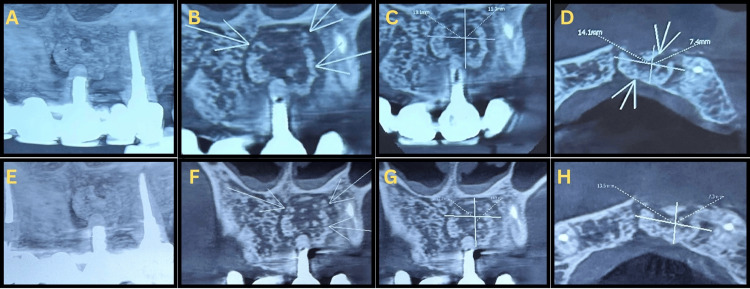
Six-month and nine-month CBCT evaluation demonstrating progressive osseous healing (A–D) Six-month follow-up: (A) 3D reconstruction showing reduction in the radiolucent area and partial cortical restoration; (B, C) coronal sections showing early trabecular reorganization and decreased lesion height; (D) axial section showing reduced lesion dimensions (≈13.1 × 11.3 × 7.4 mm) with residual cortical discontinuity. (E–H) Nine-month follow-up: (E) 3D reconstruction showing near-complete healing and cortical continuity; (F, G) coronal sections showing dense bone fill and resolution of the periapical defect; (H) axial section confirming reformation of the trabecular pattern and cortical boundary (≈12.3 × 10.9 × 7.3 mm). Arrows indicate areas of periapical healing.

A three-month CBCT was obtained for qualitative assessment only; numeric measurements were recorded at pre-op, six-month, and nine-month time points and are reported accordingly. Interim CBCTs at six and nine months were taken as part of the institutional follow-up protocol to monitor bone graft consolidation and cortical regeneration. At six months, CBCT (Figure 2, Panel ​​​​​​​A) demonstrated a reduction of lesion dimensions (≈13.1 × 11.3 × 7.4 mm) with trabecular bridging and early bone regeneration. At nine months, the lesion had further decreased (≈12.3 × 10.9 × 7.3 mm) with re-established cortical continuity and dense bone formation (Figure 2, Panels B and C). The FPD remained intact, and the patient reported full resolution of discomfort.

The described surgical protocol was customized to the patient’s prosthetic and anatomical constraints, following standard clinical principles of case-specific microsurgical management.

Tool/instrument usage statement

No proprietary tools, scoring systems, or licensed software were used in this study. All clinical instruments (CBCT unit, Biodentine®, APRF preparation kits, and ultrasonic units) are standard, commercially available, and used under routine institutional clinical protocols.

## Discussion

This case highlights the importance of integrating prosthetic, anatomical, and financial factors when formulating endodontic treatment plans [9]. In teeth supporting an FPD, orthograde retreatment can endanger abutments and compromise aesthetics [10]. As highlighted by Setzer and Kratchman (2022), surgical endodontic intervention can be the preferred treatment option in situations where orthograde retreatment is limited by prosthetic or structural constraints, allowing for predictable healing while preserving existing restorations [6].

The Luebke-Oschenbein flap was selected for its multiple advantages over conventional full-thickness or triangular mucoperiosteal flaps. It preserves the marginal gingiva and interdental papillae, thereby maintaining esthetics in the anterior region and avoiding gingival recession around prosthetic crowns. The submarginal incision design provides excellent surgical access while minimizing postoperative discomfort and edema, which is particularly beneficial when the tooth serves as an abutment beneath a fixed prosthesis [8]. In comparison, triangular or rectangular flaps may lead to greater marginal trauma, compromised blood supply, and recession, making them less suitable in prosthetically constrained cases. This approach thus balanced surgical visibility, tissue preservation, and esthetic integrity. Biodentine provides superior sealing and biocompatibility compared to traditional retrograde materials [11]. Regenerative adjuncts such as APRF accelerate bone healing through growth factor release. CBCT provides an accurate 3D evaluation of bone density and defect fill [6]. In our case, volumetric reduction and trabecular reorganization were already visible at six months, with further consolidation by nine months [12].

Although the surgical workflow described in this report is reproducible, endodontic microsurgical management must always be individualized. The exact choice of flap design, regenerative adjuncts, and postoperative protocol depends on multiple factors such as the extent of periapical pathology, bone quality, prosthesis type, and the patient’s systemic condition. Therefore, this case represents one clinical adaptation within a broad therapeutic spectrum, and treatment should always be planned based on the patient’s unique anatomical and restorative considerations.

Although quantitative bone density analysis could not be performed, progressive linear reduction in lesion dimensions and trabecular reorganization on CBCT served as objective indicators of osseous healing. The Luebke-Oschenbein flap was selected not only for aesthetic preservation but also for its ability to provide excellent surgical access with minimal risk of gingival recession or marginal trauma. Potential complications such as graft contamination, apical leakage, or soft-tissue dehiscence were prevented through aseptic technique, adequate flap design, ultrasonic retropreparation, and secure closure under tension-free conditions. Limitations include the single-case design and limited generalizability. Potential complications such as apical leakage, reinfection, or incomplete graft integration must be considered when extrapolating these findings.

## Conclusions

When orthograde retreatment is not feasible due to prosthetic limitations or apical obstructions, surgical endodontics employing bioactive and regenerative materials offers a predictable alternative with minimal morbidity. The use of a Luebke-Oschenbein flap ensured tissue preservation and aesthetic integrity, while Biodentine, APRF, and bone grafting facilitated rapid periapical bone regeneration, as confirmed radiographically by CBCT. This case highlights the importance of case-specific microsurgical planning and integration of regenerative adjuncts to optimize healing outcomes. Long-term follow-up further reinforces that endodontic microsurgery, when combined with contemporary biomaterials, can achieve complete functional and structural restoration even in prosthetically constrained scenarios.
